# A quantitative genetic and epigenetic model of complex traits

**DOI:** 10.1186/1471-2105-13-274

**Published:** 2012-10-26

**Authors:** Zhong Wang, Zuoheng Wang, Jianxin Wang, Yihan Sui, Jian Zhang, Duanping Liao, Rongling Wu

**Affiliations:** 1 Siyang Science and Technology Station, Yuanpeng Institute of Genome, Nantong, Jiangsu, 226019, China; 2 Department of Public Health Sciences, Penn State College of Medicine, Hershey, PA, USA; 3 Division of Biostatistics, Yale University, New Haven, CT, 06510, USA; 4 Center for Computational Biology, Beijing Forestry University, Beijing, 100083, China

## Abstract

**Background:**

Despite our increasing recognition of the mechanisms that specify and propagate epigenetic states of gene expression, the pattern of how epigenetic modifications contribute to the overall genetic variation of a phenotypic trait remains largely elusive.

**Results:**

We construct a quantitative model to explore the effect of epigenetic modifications that occur at specific rates on the genome. This model, derived from, but beyond, the traditional quantitative genetic theory that is founded on Mendel’s laws, allows questions concerning the prevalence and importance of epigenetic variation to be incorporated and addressed.

**Conclusions:**

It provides a new avenue for bringing chromatin inheritance into the realm of complex traits, facilitating our understanding of the means by which phenotypic variation is generated.

## Background

Systematic or stochastic changes in chromatin states, such as DNA methylation, chromatin remodeling, histone modification and RNA interference, have been thought to provide an additional driving force for phenotypic variation in complex traits and diseases [1-9]. Different chromatin states, called epialleles, that occur in the same sequence allele cannot be captured by an analysis based on DNA sequence alone [10]. With the increasing availability of epigenome technologies, there has been an unprecedented opportunity to understand the role of epiallelic variants in maintaining and inducing functional variation for organisms to better buffer against environmental perturbations. This hence entails the development of quantitative models that can enable our knowledge about the amount and pattern of quantitative variation determined by epialleles. By integrating with linkage or association mapping strategies, these models can retrieve epigenetic variation that cannot be estimated presently [10-13].

There have been several publications on methodological development for epigenetic detection [14-17]. Johannes and Colome-Tatche [16] proposed an experimental approach for estimating epigenetic variation in experimental crosses derived from epigenomically perturbed isogenic lines. This approach is powered to model the effects of epiallelic instability, recombination, parent-of-origin effects, and transgressive segregation on phenotypic variation across generations. Tal et al. [15] derived an expression form for covariances between relatives due to epigenetic transmissibility. A statistical model based on multiple testing procedures has been developed to identify the genomic regions of epigenetic variability among different individuals from genome-wide DNA methylation data [18]. These model developments, in a combination with empirical studies, can be used to test the hypothesis that epigenetic variation arising from chromatin modifications of DNA directly or indirectly is an important contributor to the missing heritability [17,19].

Despite these advances, we are still unclear how much of the phenotypic variation is contributed by epigenetic modifications and, more importantly, through which way epialleles trigger their effects on phenotypic values. The motivation of this article is to develop a quantitative model for estimating and testing the contribution of epigenetic variants to quantitative trait variation. The model allows the prediction of how much genetic variation is produced through a change in the rate of occurrence of epigenetic mutation and the effect of epigenetic factors in a natural population. We particularly discuss how the epigenetic effect interacts with other genetic effects, such as additive and dominant, to affect phenotypic traits. By implementing it into genome-wide association studies [19], the model proposed provides useful guidance for designing efficient and effective molecular experiments to characterize a comprehensive picture of the epigenetic variation of complex traits or diseases in different organisms.

## Model

### Occurrence rate of methylation

Consider an epigenetic study population of *n* individuals that are randomly drawn from a natural population, in which a nucleotide site, with two alleles *A*_1_ and *A*_2_, is thought to affect a phenotypic trait. Let *p* and *q* (*p* + *q* = 1) denote the allele frequencies of *A*_1_ and *A*_2_ in the natural population at Hardy-Weinberg equilibrium (HWE), respectively. The genotypic frequencies of *A*_1_*A*_1_, *A*_1_*A*_2_, and *A*_2_*A*_2_ at the nucleotide site studied are expressed as *p*^2^, 2*pq*, and *q*^2^, respectively [20,21].

At the nucleotide site studied, some cytosines within a CpG dinucleotide are methylated by adding a methyl group to the 5 position of the cytosine pyrimidine ring. With no loss of generality, allele *A*_1_ is a cytosine which is, if any, methylated into a new “allele” called the epiallele, denoted as *A*_*e*_, at a rate *u*. After DNA methylation, the population frequencies of non-methylated *A*_1_ allele, epiallele *A*_*e*_ and allele *A*_2_ are (1 – *u*)*p*, *up*, and *q*, respectively. Current technologies allow the distinction of epialleles from non-methylated alleles. The process of methylation and the resulting frequencies of six distinguishable genetic and epigenetic types are expressed as

(1) $$\begin{array}{c} \text{Genotype}/\text{epigenotype}\\ A_{1}A_{1}\Rightarrow \{\begin{array}{l} A_{1}A_{1}\;\text{No methylation}\\ A_{1}A_{e}\;\text{One methylation}\\ A_{e}A_{e}\;\text{Two methylations} \end{array}\\ \begin{array}{c} A_{1}A_{2}\Rightarrow \{\begin{array}{l} A_{1}A_{2}\;\text{No methylation}\\ A_{2}A_{e}\;\text{One methylation} \end{array}\\ A_{2}A_{2\;}Þ\;A_{2}A_{2}\;\text{No methylation} \end{array} \end{array}\;\begin{array}{c} \text{Frequency}\\ \left\{ \begin{array}{l} {\left(1-u\right)}^{2}p^{2}+D_{12}+D_{1e}\\ 2u(1-u)p^{2}-2D_{1e}\\ u^{2}p^{2}+D_{1e}+D_{2e} \end{array}\right.\\ \begin{array}{c} \left\{ \begin{array}{l} 2(1-u)pq-2D_{12}\\ 2upq-2D_{2e} \end{array}\right.\\ q^{2}+D_{12}+D_{2e} \end{array} \end{array}\;\begin{array}{c} \text{Observation}\\ \left\{ \begin{array}{l} n_{11}\\ n_{1e}\\ n_{\mathrm{ee}} \end{array}\right.\\ \begin{array}{c} \left\{ \begin{array}{l} n_{12}\\ n_{2e} \end{array}\right.\\ n_{22} \end{array} \end{array}$$

where *D*_12_, *D*_1*e*_, and *D*_2*e*_ are the coefficients of Hardy-Weinberg disequilibrium (HWD) due to a non-random association between alleles *A*_1_ and *A*_2_, between allele *A*_1_ and epiallele *A*_*e*_, and between allele *A*_2_ and epiallele *A*_*e*_, respectively. It is possible that the previous equilibrium of the population is violated by DNA methylation, leading to the HWD quantified by *D*_12_, *D*_1*e*_, and *D*_2*e*_. Thus, the genotype and epigenotype frequencies may be determined by allele and epiallele frequencies and HWD coefficients.

Let *n*_11_, *n*_1*e*_, *n*_*ee*_, *n*_12_, *n*_2*e*_, and *n*_22_ (*n*_11_+*n*_1*e*_+*n*_*ee*_+*n*_12_+*n*_2*e*_+*n*_22_ = *n*) denote the observations of the corresponding genotypes/epigenotypes (1) in the study population. Based on the frequencies of these genotypes/epigenotypes, we formulate a polynomial likelihood from which to obtain the maximum likelihood estimates (MLEs) of the allele frequencies, the occurrence frequency of methylation, and HWD using

(2) $$\hat{p}=\frac{n_{11}+n_{1e}+n_{\mathrm{ee}}+\frac{1}{2}\left(n_{12}+n_{2e}\right)}{n}$$

(3) $$\hat{u}=\frac{n_{\mathrm{ee}}+\frac{1}{2}\left(n_{1e}+n_{2e}\right)}{n_{11}+n_{1e}+n_{\mathrm{ee}}+\frac{1}{2}\left(n_{12}+n_{2e}\right)}$$

(4) $$\hat{q}=\frac{n_{22}+\frac{1}{2}\left(n_{12}+n_{2e}\right)}{n}$$

(5) $${\hat{D}}_{1e}=\hat{u}\left(1-\hat{u}\right){\hat{p}}^{2}-\frac{n_{1e}}{2n}$$

(6) $${\hat{D}}_{2e}=\hat{u}\hat{p}\hat{q}-\frac{n_{2e}}{2n}$$

(7) $${\hat{D}}_{12}=\left(1-\hat{u}\right)\hat{p}\hat{q}-\frac{n_{12}}{2n}$$

We are interested in investigating whether there is significant occurrence of DNA methylation at the nucleotide site. This can be tested by formulating a null hypothesis, H_0_: *u* = 0, vs. an alternative hypothesis, H_0_: *u* ≠ 0, under each of which the likelihoods (L_0_ and L_1_) are calculated, respectively. However, because the *u* value in the H_0_ lies on the boundary of parameter space, the log-likelihood ratio calculated,

$$\text{LR}=-2\left({\text{log L}}_{0}–{\text{log L}}_{1}\right)\text{,}$$

may not follow a standard chi-square distribution. Self and Liang [22] showed that the null distribution of the LR test statistic is a mixture of projections of chi-square variables onto surfaces, with the weights of mixtures that can be derived analytically only in special cases. By establishing the asymptotic null and alternative distributions of quasi-likelihood ratio, rescaled quasi-likelihood ratio, Wald, and score tests, Andrews [23] suggested the use of these test statistics to test the boundary value of a model parameter. While the first three test statistics are easy to compute, the score test is more difficult by deriving the first and second-order derivatives of the alternative log-likelihood.

Similar tests can be performed for individual HWD, *D*_1*e*_, *D*_2*e*_, or *D*_12_, or their combinations, by formulating the null hypotheses, respectively. Under the alternative hypothesis H_1_ associated with each null hypothesis considered, the likelihood is calculated. The LR value calculated is thought to be asymptotically chi-square distributed with the degree of freedom equal to the difference in the number of parameters to be estimated between the alternative and null hypotheses.

### Genetic and epigenetic effect

We assume that the study population is investigated under a uniform condition so that the phenotypic variation can be simply partitioned into genetic/epigenetic components and errors. There are only three genotypes, *A*_1_*A*_1_, *A*_1_*A*_2_, and *A*_2_*A*_2_, prior to DNA methylation. Let *a* denote the additive effect of the nucleotide site due to the substitution of allele *A*_1_ by *A*_2_ or vice versa and *d* denote the dominant effect due to the interaction between the two alleles. The values of three genotypes are diagrammed over an axis as follows:

(8) $$\begin{array}{l} \text{Genotype}\;A_{2}A_{2}\;A_{1}A_{2\;}A_{1}A_{1}\\ \text{Genotypic value}\;\mu –a\;\mu \;\mu +d\;\mu +a\\ \text{Net genotypic value}\;–a\;0\;d\;a\\ \;\overset{―}{\text{Origin}} \end{array}$$

As described above, allele *A*_1_ is assumed to be methylated into the epiallele *A*_*e*_. The values of six distinguishable genetic and epigenetic types are expressed as

(9) $$\begin{array}{c} \text{Genotype}/\text{epigenotype}\\ A_{1}A_{1}\Rightarrow \{\begin{array}{l} A_{1}A_{1}\;\text{No methylation}\\ A_{1}A_{e}\;\text{One methylation}\\ A_{e}A_{e}\;\text{Two methylations} \end{array}\\ \begin{array}{c} A_{1}A_{2}\Rightarrow \{\begin{array}{l} A_{1}A_{2}\;\text{No methylation}\\ A_{2}A_{e}\;\text{One methylation} \end{array}\\ A_{2}A_{2\;}Þ\;A_{2}A_{2}\;\text{No methylation} \end{array} \end{array}\;\begin{array}{c} \text{Expected Value}\\ \left\{ \begin{array}{l} \mu +a_{1}\\ \mu +\frac{1}{2}(a_{1}+a_{e})+d_{1e}\\ \mu +a_{e} \end{array}\right.\\ \begin{array}{c} \left\{ \begin{array}{l} \mu -\frac{1}{2}a_{e}+d_{12}\\ \mu -\frac{1}{2}a_{1}+d_{2e} \end{array}\right.\\ \mu –a_{1}–a_{e} \end{array} \end{array}\;\begin{array}{c} \text{Estimated Value}\\ \left\{ \begin{array}{l} \sum_{i=1}^{n_{11}}y_{i}/n_{11}\\ \sum_{i=1}^{n_{1e}}y_{i}/n_{1e}\\ \sum_{i=1}^{n_{\mathrm{ee}}}y_{i}/n_{\mathrm{ee}} \end{array}\right.\\ \begin{array}{c} \left\{ \begin{array}{l} \sum_{i=1}^{n_{12}}y_{i}/n_{12}\\ \sum_{i=1}^{n_{2e}}y_{i}/n_{2e} \end{array}\right.\\ \sum_{i=1}^{n_{22}}y_{i}/n_{22} \end{array} \end{array}$$

where the genotypic value of the trait is decomposed into different components, i.e., the overall mean (*μ*), the additive effects due to the substitution of allele *A*_1_ (*a*_1_) and epiallele *A*_*e*_ by allele *A*_2_ (*a*_*e*_), and the dominance effects due to the interaction between allele *A*_1_ and epiallele *A*_*e*_ (*d*_1*e*_), between allele *A*_1_ and allele *A*_2_ (*d*_12_) and between allele *A*_2_ and epiallele *A*_*e*_ (*d*_2*e*_).

Let *y*_*i*_ denote the phenotypic value of the trait for individual *i* (*i* =1, …, *n*) in the study population. The MLEs of the genotypic value for each genotype/epigenotype can be obtained by simply taking its mean over all individuals belonging to this genotype/epigenotye (9). The genetic and epigenetic effects can be estimated by solving a group of regular equations for the genotypic values (9), i.e.,

(10) $${\hat{a}}_{1}=\frac{1}{3}\left[\frac{2\sum_{i=1}^{n_{11}}y_{i}}{n_{11}}-\left(\frac{\sum_{i=1}^{n_{\mathrm{ee}}}y_{i}}{n_{\mathrm{ee}}}+\frac{\sum_{i=1}^{n_{22}}y_{i}}{n_{22}}\right)\right]$$

(11) $${\hat{a}}_{e}=\frac{1}{3}\left[\frac{2\sum_{i=1}^{n_{\mathrm{ee}}}y_{i}}{n_{\mathrm{ee}}}-\left(\frac{\sum_{i=1}^{n_{11}}y_{i}}{n_{11}}+\frac{\sum_{i=1}^{n_{22}}y_{i}}{n_{22}}\right)\right]$$

(12) $${\hat{d}}_{1e}=\frac{\sum_{i=1}^{n_{1e}}y_{i}}{n_{1e}}-\frac{1}{2}\left(\frac{\sum_{i=1}^{n_{11}}y_{i}}{n_{11}}+\frac{\sum_{i=1}^{n_{\mathrm{ee}}}y_{i}}{n_{\mathrm{ee}}}\right)$$

(13) $${\hat{d}}_{2e}=\frac{\sum_{i=1}^{n_{2e}}y_{i}}{n_{2e}}-\frac{1}{2}\left(\frac{\sum_{i=1}^{n_{22}}y_{i}}{n_{22}}+\frac{\sum_{i=1}^{n_{\mathrm{ee}}}y_{i}}{n_{\mathrm{ee}}}\right)$$

(14) $${\hat{d}}_{12}=\frac{\sum_{i=1}^{n_{12}}y_{i}}{n_{12}}-\frac{1}{2}\left(\frac{\sum_{i=1}^{n_{11}}y_{i}}{n_{11}}+\frac{\sum_{i=1}^{n_{22}}y_{i}}{n_{22}}\right)$$

Each of these effects (10) – (14) can be tested by the log-likelihood ratio approach. For an epigenetic study, we are more interested in testing the epigenetic effect of the nucleotide site *a*_*e*_ and dominant effects due to the interactions between the alleles and epiallele *d*_1*e*_ and *d*_2*e*_. The log-likelihood ratio test statistics for each hypothesis test is thought of being asymptotically chi-square distributed with the degree of freedom equal to the difference in the number of parameters to be estimated between the alternative and null hypotheses.

### Genetic and epigenetic variation

We first give the genetic variance explained by the nucleotide site studied prior to DNA methylation. By defining a new parameter called the average effect *α* = *a* + (*qp*)*d*[20], we derived the overall genetic variance of the trait due to this site as

(15) $$\sigma_{g}^{2}=2pq\alpha^{2}+{\left(2pqd\right)}^{2}\equiv \sigma_{a}^{2}+\sigma_{d}^{2}$$

where *σ*_*a*_^2^ = 2*pqα*^2^ is the additive genetic variance depending on both *a* and *d*, and *σ*_*d*_^2^ = (2*pqd*)^2^ is the dominant genetic variance only depending on *d*. Both additive and dominance variances are affected by the relative magnitudes of allele frequencies *p* and *q*. These two variances reach their maximums when two alternative alleles *A*_1_ and *A*_2_ occur at the same frequency.

In what follows, we model how the epigenetic change contributes to the genetic variance of a complex trait based on the frequencies (1) and values of genotypes/epigenotypes (9). The total genetic variation among the six genotypes/epigenotypes is derived as

(16) $$\begin{array}{l} \sigma_{G}^{2}=a_{1}^{2}\left[{\left(1-u\right)}^{2}p^{2}+D_{12}+D_{1e}\right]+a_{e}^{2}\left[u^{2}p^{2}+D_{1e}+D_{2e}\right]\\ \;+{\left(a_{1}+a_{e}\right)}^{2}\left[q^{2}+D_{12}+D_{2e}\right]+{\left[\frac{1}{2}\left(a_{1}+a_{e}\right)+d_{1e}\right]}^{2}\\ \;\times \left[2u\left(1-u\right)p^{2}-2D_{1e}\right]+{\left[-\frac{1}{2}a_{1}+d_{2e}\right]}^{2}\\ \;\times \left[2upq-2D_{2e}\right]+{\left[-\frac{1}{2}a_{e}+d_{12}\right]}^{2}\\ \;\times \left[2\left(1-u\right)pq-2D_{12}\right]-m^{2} \end{array}$$

where *m* is the population mean expressed as

$$\begin{array}{l} m=a_{1}\left[\left(1-u\right)p-q\right]+a_{e}\left(up-q\right)\\ \;+2d_{1e}\left[u\left(1-u\right)p^{2}-D_{1e}\right]+2d_{2e}\left(upq-D_{2e}\right)\\ \;+2d_{12}\left[\left(1-u\right)pq-D_{12}\right] \end{array}$$

It can be seen from equation (16) that the total genetic variance includes 15 different parts, i.e.,

$$\begin{array}{l} \sigma_{G}^{2}=\sigma_{a_{1}}^{2}\;\text{Additive effect of the original alleles prior to methylation}\\ \;+\sigma_{a_{e}}^{2}\;\text{Additive effect of the epiallele}\\ \;+\sigma_{d_{1e}}^{2}\;\text{Domiant effect between the original allele and epiallele}\\ \;+\sigma_{d_{2e}}^{2}\;\text{Domiant effect between the original allele and epiallele}\\ \;+\sigma_{d_{12}}^{2}\;\text{Domiant effect between the original alleles}\\ \;+\sigma_{a_{1}\times a_{e}}^{2}\;\text{Multiplicative additive}\;\times \;\text{additive effect involving the epiallele}\\ \;+\sigma_{a_{1}\times d_{1e}}^{2}\;\text{Multiplicative additive}\;\times \;\text{dominant effect involving the epiallele}\\ \;+\sigma_{a_{1}\times d_{2e}}^{2}\;\text{Multiplicative additive}\;\times \;\text{dominant effect involving the epiallele}\\ \;+\sigma_{a_{1}\times d_{12}}^{2}\;\text{Multiplicative additive}\;\times \;\text{dominant effect with no epiallele}\\ \;+\sigma_{a_{e}\times d_{1e}}^{2}\;\text{Multiplicative additive}\;\times \;\text{dominant effect involving the epiallele}\\ \;+\sigma_{a_{e}\times d_{2e}}^{2}\;\text{Multiplicative additive}\;\times \;\text{dominant effect involving the epiallele}\\ \;+\sigma_{a_{e}\times d_{12}}^{2}\;\text{Multiplicative additive}\;\times \;\text{dominant effect involving epiallele}\\ \;+\sigma_{d_{1e}\times d_{2e}}^{2}\;\text{Multiplicative dominant}\;\times \;\text{additive effect involving the epiallele}\\ \;+\sigma_{d_{1e}\times d_{12}}^{2}\;\text{Multiplicative additive}\;\times \;\text{dominant effect involving the epiallele}\\ \;+\sigma_{d_{2e}\times d_{12}}^{2}\;\text{Multiplicative additive}\;\times \;\text{dominant effect involving the epiallele} \end{array}$$

Here, we define a new heritability, called the epigenetic heritability, which describes the proportion of the phenotypic variance explained by the effect of the epiallele and its interactions with the other effects, expressed as

(17) $$H_{e}^{2}=\frac{\sigma_{G}^{2}-\sigma_{a_{1}}^{2}-\sigma_{d_{12}}^{2}-\sigma_{a_{1}\times d_{12}}^{2}}{\sigma_{P}^{2}}$$

Also, we use the proportion of the epigenetic variance to the total genetic variance to describe the relative contribution of epigenetic methylation to the overall genetic variance, expressed as

(18) $$R_{e}^{2}=\frac{\sigma_{G}^{2}-\sigma_{a_{1}}^{2}-\sigma_{d_{12}}^{2}-\sigma_{a_{1}\times d_{12}}^{2}}{\sigma_{G}^{2}}$$

These two parameters can be used to assess the contribution of DNA methylation to the total phenotypic variation of a quantitative trait.

### Numerical analysis

In this section, we performed numerical analyses to investigate how epigenetic marks contribute to the heritability of a complex trait. The occurrence of epigenetic marks is described by population genetic parameters including the occurrence rate of the epiallele and its Hardy-Weinberg disequilibria with unmarked alleles. The effect of epigenetic marks can be specified by quantitative genetic parameters including the epigenetic effect of the epiallele and its interactions with other effects. As analyzed above, population genetic parameters (*p*, *q*, *u*, *D*_1*e*_, *D*_2*e*_, *D*_12_) and quantitative genetic parameters (*a*_1_, *a*_*e*_, *d*_1*e*_, *d*_2*e*_, *d*_12_) contribute to the genetic variance in a complex way (16). We will analyze the contribution of epigenetic marks by separately investigating how these population and quantitative genetic parameters affect *R*_*e*_^2^.

### Population genetic effect

Suppose there is a study population in which methylated sites are observed for a phenotypic trait. Consider a nucleotide site with two alleles *A*_1_ and *A*_2_, one of which, say *A*_1_, is methylated at a rate *u* (*u* takes any value in [0,1]). This methylation may violate the previous HWE assumption. Based on a simple algebraic analysis, we obtain the intervals of *D*_1*e*_, *D*_2*e*_ and *D*_12_ as follows:

$$\begin{array}{l} –\frac{1}{2}\left[{\left(1–u\right)}^{2}p^{2}+u^{2}q^{2}+\left(D_{12}+D_{2e}\right)\right]\leq D_{1e}\leq \left(1–u\right)p^{2}\\ –\frac{1}{2}\left[u^{2}p^{2}+q^{2}+\left(D_{1e}+D_{12}\right)\right]\leq D_{2e}\leq upq\\ –\frac{1}{2}\left[{\left(1–u\right)}^{2}p^{2}+q^{2}+\left(D_{1e}+D_{2e}\right)\right]\leq D_{12}\leq \left(1–u\right)pq \end{array}$$

**Figure 1:**
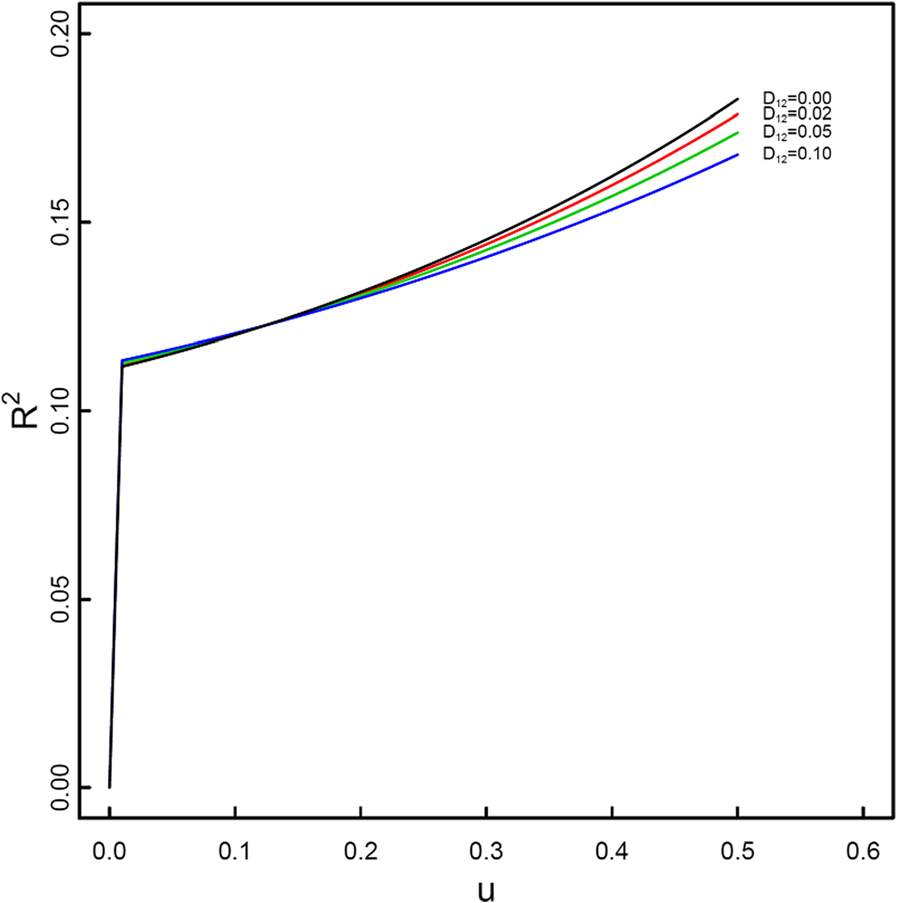
**Change of the proportion of the epigenetic variance over the total genetic variance (*R***_***e***_^**2**^**) as a function of the occurrence rate of methylation in a natural population.** The total and epigenetic genetic variances are calculated by assuming population genetic parameters (*p*, *q*, *u*, *D*_1*e*_, *D*_2*e*_, *D*_12_) ≡ (0.4, 0.6, *u*, 0.05, 0.05, *D*_12_) (allowing *u* and *D*_12_ to change) and quantitative genetic parameters (*a*_1_, *a*_*e*_, *d*_1*e*_, *d*_2*e*_, *d*_12_) ≡ (0.4, 0.05, 0.05, 0.05, 0.05).

**Figure 2:**
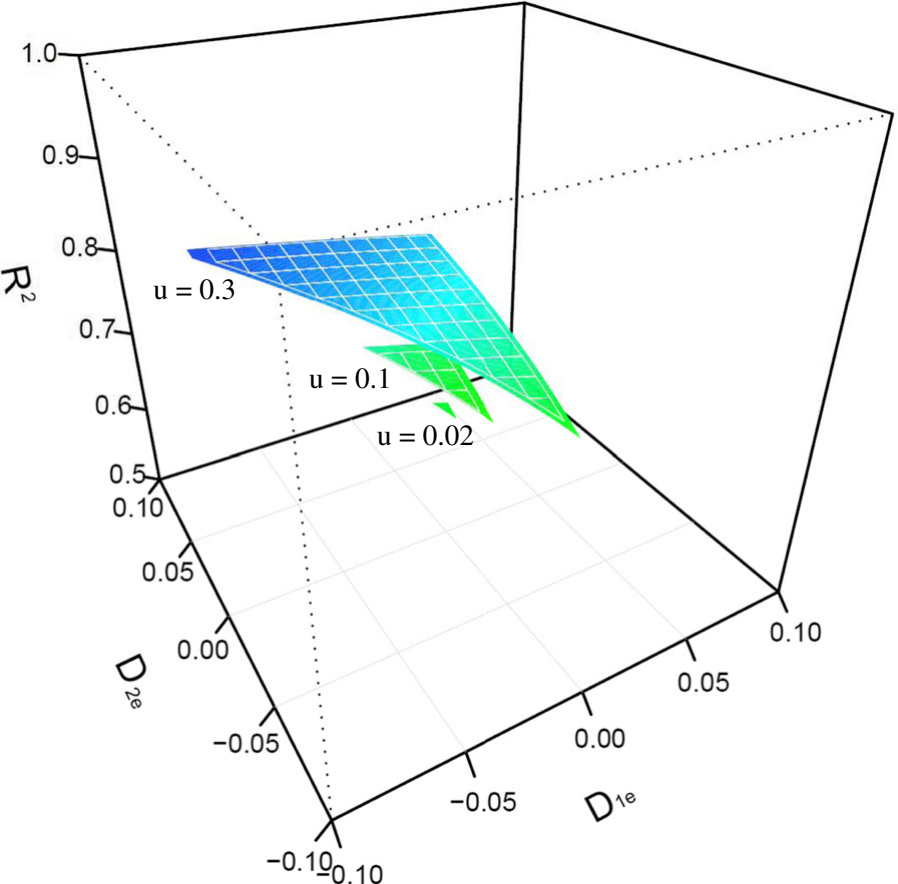
**Change of the proportion of the epigenetic variance over the total genetic variance (*R***_***e***_^**2**^**) as a function of Hardy-Weinberg disequilibrium (HED) coefficients formed between the original allele and epiallele in a natural population after DNA methylation.** The total and epigenetic genetic variances are calculated by assuming population genetic parameters (*p*, *q*, *u*, *D*_1*e*_, *D*_2*e*_, *D*_12_) ≡ (0.4, 0.6, *u*, *D*_1*e*_, *D*_2*e*_, 0) (allowing *u*, *D*_1*e*_, and *D*_2*e*_ to change) and quantitative genetic parameters (*a*_1_, *a*_*e*_, *d*_1*e*_, *d*_2*e*_, *d*_12_) ≡ (0.4, 0.05, 0.05, 0.05, 0.05).

Because of DNA methylation, the change of the genetic variance explained by the site takes place. By fixing quantitative genetic parameters, we quantitatively examined the impacts of different occurrence rates of methylation and different HWD coefficients on the epigenetic ariance. A small value of occurrence rate may lead to the formation of substantial epigenetic variance, although this phenomenon depends on the disequilibrium degree of association between two original alleles produced following methylation (Figure 1). The epigenetic variance is also positively associated with the degree of disequilibrium for the unmarked alleles and epiallele (Figure 2).

### Quantitative genetic effect

**Figure 3:**
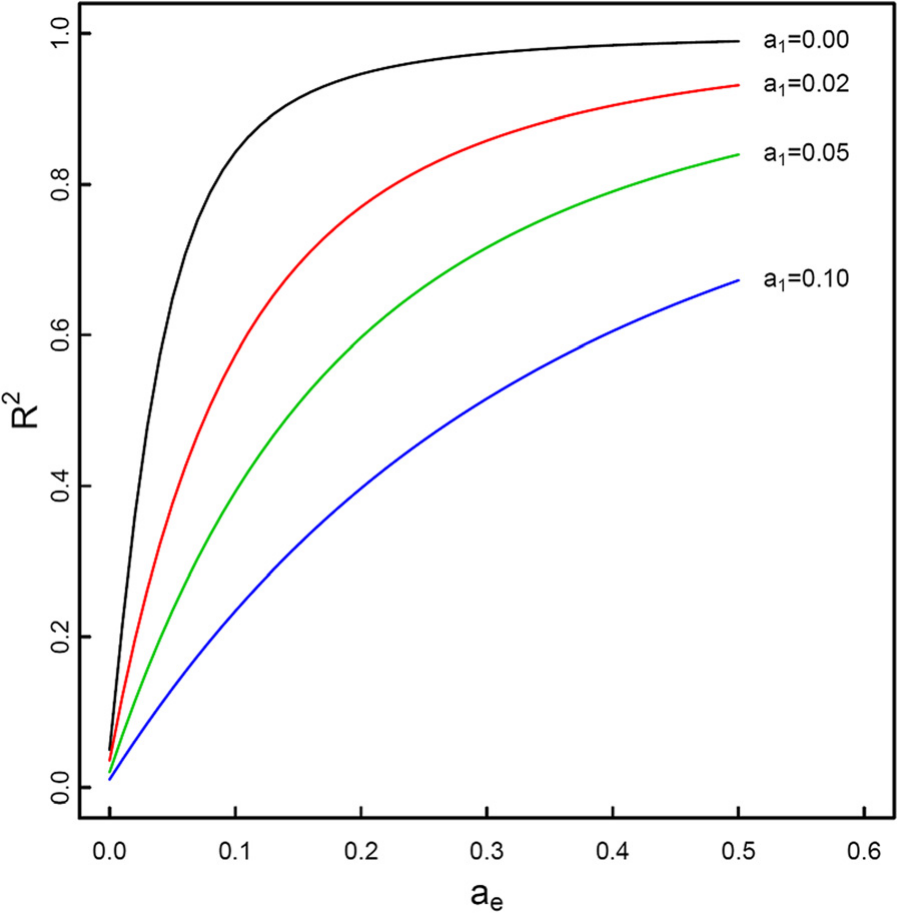
**Change of the proportion of the epigenetic variance over the total genetic variance (*R***_***e***_^**2**^**) as a function of the additive genetic effect due to the substitution of the original allele by the epiallele.** The total and epigenetic genetic variances are calculated by assuming population genetic parameters (*p*, *q*, *u*, *D*_1*e*_, *D*_2*e*_, *D*_12_) ≡ (0.4, 0.6, 0.2, 0, 0, 0) and quantitative genetic parameters (*a*_1_, *a*_*e*_, *d*_1*e*_, *d*_2*e*_, *d*_12_) ≡ (*a*_1_, *a*_*e*_, 0.05, 0.05, 0.05) (allowing *a*_1_ and *a*_*e*_ to change).

**Figure 4:**
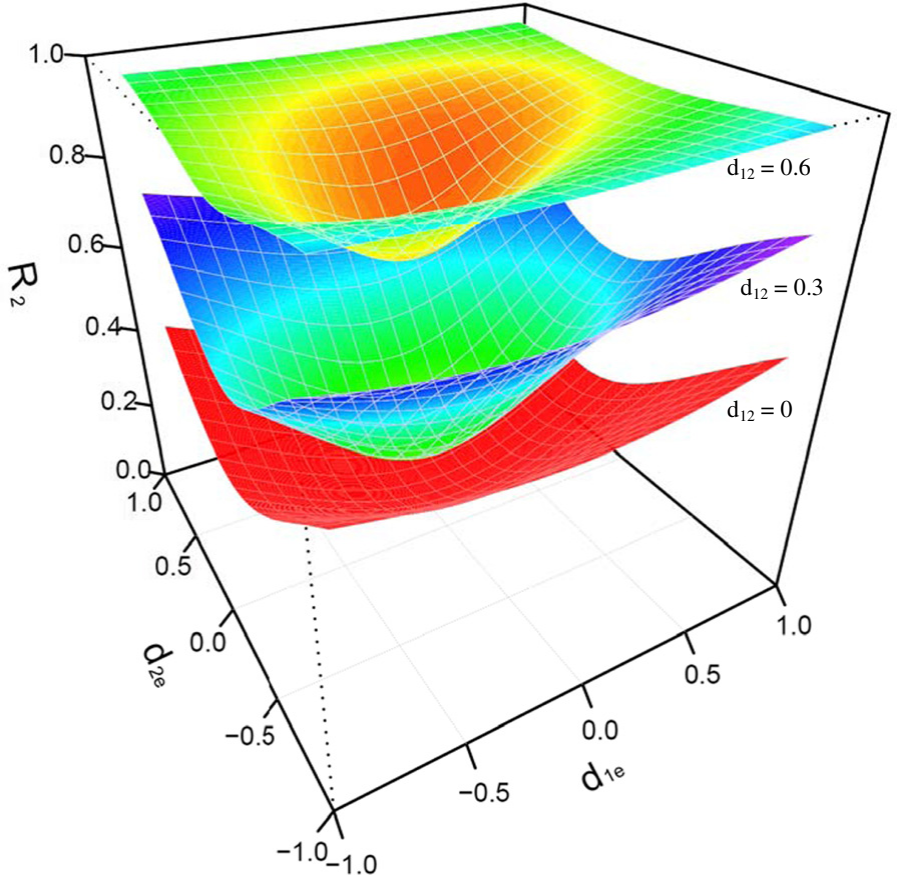
**Change of the proportion of the epigenetic variance over the total genetic variance (*R***_***e***_^**2**^**) as a function of the dominant genetic effect due to the interaction between the original allele and epiallele.** The total and epigenetic genetic variances are calculated by assuming population genetic parameters (*p*, *q*, *u*, *D*_1*e*_, *D*_2*e*_, *D*_12_) ≡ (0.4, 0.6, 0.2, 0.01, 0.01, 0) and quantitative genetic parameters (*a*_1_, *a*_*e*_, *d*_1*e*_, *d*_2*e*_, *d*_12_) ≡ (0.08, 0.12, *d*_1*e*_, *d*_2*e*_, *d*_12_) (allowing *d*_1*e*_, *d*_2*e*_ and *d*_12_ to change).

By fixing population genetic parameters, the influence of genetic effects triggered by the epiallele was investigated. A small value of the additive effect *a*_*e*_ formed by the epiallele brings about considerable epigenetic variance (Figure 3). This influence increases with increasing *a*_*e*_ values. The epigenetic variance is also remarkably affected by the dominant effect between the original alleles and epiallele (Figure 4). It is clear that these effect parameters contribute to the epigenetic variance also through their complex interactions.

### Computer simulation

Our model allows the estimation and test of epigenetic effects. We carried out simulation studies to examine the statistical properties of the model. A study population was simulated by assuming a set of population and quantitative genetic parameters and a normally distributed residual error with mean zero and variance scaled under a range of trait heritabilities. As expected, the estimation precision increases with increasing sample size and heritability. A sample size 400 is sufficient to provide reasonable estimates of all population genetic parameters (Table 1). Note that the estimation precision of the population parameters does not rely on the size of heritability. In general, the reasonable estimation of quantitative genetic parameters, especially dominant genetic effects, needs a much larger sample size, say 1000 (Table 1). As expected, the estimation precision of genetic effects is sensitive to heritability. In practice, every effort should be given to precisely measure the phenotypic trait, aimed to increase the level of heritability.

**Table 1:** **MLEs of population and quantitative genetic parameters from simulated data with different heritabilities (*H***^**2**^**) and sample sizes (*n*)**

	**True**	***H***^**2**^**= 0.05**		***H***^**2**^**= 0.1**		***H***^**2**^**= 0.2**	
		**MLE SD**		**MLE SD**		**MLE SD**	
*n*=400	0.1	0.099 (0.019)		0.100 (0.017)		0.099 (0.020)	
*u*							
*p*	0.4	0.399 (0.020)		0.400 (0.022)		0.403 (0.018)	
*D*_12_	0.01	0.011 (0.010)		0.008 (0.011)		0.009 (0.012)	
*D*_1e_	0.01	0.010 (0.003)		0.010 (0.003)		0.010 (0.003)	
*D*_2e_	0.01	0.010 (0.004)		0.010 (0.005)		0.010 (0.004)	
*μ*	1	1.002 (0.096)		1.009 (0.066)		1.002 (0.043)	
*a*_1_	0.2	0.201 (0.113)		0.193 (0.085)		0.198 (0.049)	
*a*_*e*_	0.05	0.060 (0.181)		0.064 (0.134)		0.054 (0.080)	
*d*_12_	0.05	0.050 (0.076)		0.049 (0.055)		0.049 (0.032)	
*d*_1*e*_	0.05	−0.015 (0.485)		−0.008 (0.401)		0.010 (0.267)	
*d*_2*e*_	0.05	0.027 (0.279)		0.047 (0.171)		0.042 (0.116)	
*n*=1000	0.1	0.101 (0.014)		0.101 (0.013)		0.102 (0.013)	
*u*							
*p*	0.4	0.401 (0.012)		0.400 (0.012)		0.401 (0.011)	
*D*_12_	0.01	0.010 (0.007)		0.009 (0.007)		0.010 (0.007)	
*D*_1*e*_	0.01	0.010 (0.003)		0.010 (0.002)		0.010 (0.002)	
*D*_2*e*_	0.01	0.010 (0.003)		0.010 (0.003)		0.010 (0.003)	
*μ*	1	1.003 (0.053)		0.996 (0.039)		1.000 (0.027)	
*a*_1_	0.2	0.202 (0.067)		0.207 (0.049)		0.200 (0.031)	
*a*_*e*_	0.05	0.051 (0.099)		0.037 (0.068)		0.050 (0.050)	
*d*_12_	0.05	0.051 (0.048)		0.047 (0.035)		0.051 (0.023)	
*d*_1*e*_	0.05	0.056 (0.269)		0.046 (0.191)		0.038 (0.122)	
*d*_2*e*_	0.05	0.048 (0.158)		0.057 (0.111)		0.054 (0.081)	

The MLEs of parameters and their standard deviation (in parentheses) were calculated from 200 simulation replicates.

We also investigated the power of detecting epiallelic HWD occurrence and epigenetic effects as well as the false positive rates for epigenetic effect identification under different heritabilities and sample sizes (Table 2). Given a medium sample size 400, the model possesses adequate power (> 0.95) for the detection of small epialleli HWD coefficients, along with small false positive rates (< 0.10). The power of the model to detect epigenetic effects was calculated by testing the hypothesis, H_0_: *a*_*e*_ = *d*_1*e*_ = *d*_2*e*_ = 0 vs. H_1_: at least one of the effects in the H_0_ is not equal to zero, and comparing the resulting log-likelihood ratio test statistic with the critical threshold of a chi-square distribution with three degrees of freedom. The proportion of the number of simulation replicates that reject the null hypothesis over the total number of simulation replicates is empirically used as the power of the model. The power of epigenetic effect detection is very sensitive to the magnitude of the epigenetic effect, heritability and sample size (Table 2). When the epigenetic effect is small, the model has low power to detect it, although the power increases with increasing heritability and sample size. To detect a small epigenetic effect, a large sample size (2000 or more) is required for a precisely measured phenotype (with a large heritability). For a medium-size epigenetic effect, a sample size 1000 may be adequate for its detection if then phenotype is precisely measured. In general, the model has reasonably small false positive rates even for a medium sample size (Table 2).

**Table 2:** **The power of epigenetic-effect detection by the epigenetic model and its false positive rates (FPR) under different sample sizes (*n*) and heritabilities (*H***^**2**^**)**

	***n***	***ae *= *d***_1*e*_**= *d***_2*e*_	***H***^2^**= 0.05**	***H***^2^**= 0.1**	***H***^2^**= 0.2**
Power	400	0.05	0.055	0.090	0.160
	1000	0.05	0.090	0.125	0.355
	2000	0.05	0.115	0.205	0.630
	5000	0.05	0.215	0.415	0.975
	1000	0.1	0.085	0.255	0.780
	2000	0.1	0.265	0.525	0.975
	5000	0.1	0.495	0.950	1.00
FPR	400	0.05	0.050	0.045	0.065
	1000	0.05	0.060	0.025	0.045
	2000	0.05	0.030	0.010	0.045
	5000	0.05	0.085	0.050	0.070
	1000	0.1	0.045	0.040	0.040
	2000	0.1	0.055	0.025	0.030
	5000	0.1	0.050	0.020	0.045

### Implementing the epigenetic model into GWAS

The epigenetic model proposed can be implemented to genome-wide association studies (GWAS). In GWAS, it is likely that we have a million of methylated sites detected throughout the entire genome on a much smaller number of samples. Moreover, samples collected for human GWAS are highly heterogeneous in terms of genetic background, gender, age, race, and many other demographic characteristics. These demographic factors should be modeled as covariates. For a single methylated site, we can build a linear model to describe the phenotypic value of individual *i* by considering its multifactorial determinants, expressed as

(19) $$\begin{array}{l} y_{i}=\mu +{\xi_{i}}_{1}a_{1}+{\xi_{i}}_{2}a_{e}+{\xi_{i}}_{3}d_{1e}+{\xi_{i}}_{4}d_{2e}\\ \;+{\xi_{i}}_{5}d_{12}+\sum_{r=1}^{R}\alpha_{r}u_{\mathrm{ir}}+\sum_{s=1}^{S}\sum_{l=1}^{L_{s}}x_{\mathrm{isl}}v_{\mathrm{sl}}+e_{i} \end{array}$$

where *ξ*_*i*1_, …, *ξ*_*i*5_ are the indicator variable for subject *i* that corresponds to a specific genetic or epigenetic effect at a methylated site, *u*_*ir*_ (*r* = 1*, …, R*) is the value of the *r*th continuous covariate, such as age and BMI, for subject *i*, *α*_*r*_ is the effect of the *r*th continuous covariate, *v*_*sl*_ (*l* = 1*, …, L*_*s*_*, s* = 1*, …, S*) is the effect of the *l*th level for the *s*th discrete covariate, such as race, gender, and treatment, with ∑ _*l*=1_^*Ls*^*υsl* = 0 where *L*_*s*_ is the number of levels for the *s*th discrete covariate, *x*_*isl*_ is an indicator variable of subject *i* who receives the *l*th level of the *s*th discrete covariate, and *e*_*i*_ is a random error.

A standard multiple linear regression approach can be used to estimate all the effects described in model (19). If the test is made individually for each of the methylated sites, the significance of each effect should be adjusted by multiple comparison approaches such as Bonferroni or FDR.

Analysis of one single methylated site at a time is limited for statistical inference about a comprehensive picture of the genetic and epigenetic architecture of complex phenotypes. The best way such a picture is illustrated is to analyze all sites simultaneously. Li et al. [24] proposed a new approach by incorporating the least absolute shrinkage and selection operator (lasso) [25] to simultaneously analyze a larger number of variables using a much smaller sample size. A detailed algorithm for the Bayesian lasso has been derived [24] and can be readily implemented to GWAS aimed to identify epige-netic variants.

## Discussion

Epigenetic alternations have been increasingly recognized to play an important role in generating and maintaining quantitative genetic variation for complex phenotypes underlying physiology and diseased [6,7,9,26-28]. Preliminary estimates in plants suggest that it can account for up to 30% of the variation in commonly studied phenotypes such as height and flowering time [8]. Many theoretical models have been available to analyze the contributions of epigenetic marks to missing heritability in genome-wide association studies (GWAS) [14-18]. In this article, we extended Mendelian inheritance-based genetic principles to derive a quantitative framework by which to analyze the pattern of how DNA methylation contributes to overall genetic variance. By defining several epigenetic effect parameters, the analytical framework allows the mechanistic characterization of epigenetic actions within the quantitative genetic context.

Through numerical analysis, a small incidence of DNA methylation as well as a small effect due to methylation alternations could lead to a substantial increase of genetic variance, suggesting that epigenetic marks may be an important cause for genetic diversity in nature. Given our finding, the neglection of epigenetic variants in many current GWAS may partly explain the problem of missing heritability [17]. Simulation studies suggest that the model can provide reasonable estimates of epigenetic effect parameters with a sample size of 200 – 400, even when the trait studied has a small heritability. It should be pointed out, however, that this conclusion is based on a well-controlled study in which there are few background noises. For the GWAS in humans, the estimated genetic variation is likely to be confounded by many factors, such as population structure, heterogeneous genetic background, demographic complexity, and highly noisy phenotypic measurements among others. To remove these confounding effects from genetic and epigenetic analysis, a considerably large sample size may be needed.

The model only considers a single methylated site. However, there is no technical difficulty in extending the model to explore two or more sites at the same time which may interact with each other to produce a complex network of epistasis [29]. For two methylated sites, a total of 25 interaction parameters are formed between parameter sets each composed of (*a*_1_, *a*_*e*_, *d*_1*e*_, *d*_2*e*_, *d*_12_) for each site. In this case, an exponentially increasing sample size and more precise phenotypic measurement (aimed to increase the trait’s heritability) are needed. For the methylated population, originally existing HWE assumption may be violated in which case it is not possible to use gametic linkage disequilibria to specify the association between the two sites. Wu et al. [30] proposed a robust approach to analyze the marker-marker association by deriving a so-called zygotic linkage disequilibrium model. Wu et al.’s approach can be incorporated to identify the contribution of epigenetic marks at two sites to the overall genetic variance.

Epigenetic changes may be an adaptation to environmental perturbations [5,17,28]. Thus, it is crucial to incorporate the epigenetic model into a genotype-environment interaction study. By doing so, we can identify which and how epigenetic effects interact with the environment to determine final phenotypes so that the genetic etiology of quantitative variation can be better elucidated. In addition, there is a considerable body of evidence that epigenetic effects may transmitted from one generation to next [31,32], although other studies found the reprogramming of epigenetic effects during meiosis [5,33,34]. By embedding our epigenetic model into a family-based design, we can develop a powerful approach to test the relative importance of these two phenomena in trait control [35-37]. Traditional models analyze the inheritance of quantitative traits based on Mendel’s laws, failing to study the contribution of epigenetic modifications. In addition, many GWAS are based on a case–control study in which genotype frequencies are compared between two groups. To study the association between epigenetic effects and a particular disease, such as cancer, we can incorporate quantitative epigenetic models as described by equations (10) – (14) into a case–control framework, allowing each effect to be tested. The integration of general quantitative genetic models and a case–control design has been discussed and its statistical properties investigated through analytical derivations and computer simulations [38-40]. With these extensions, the new model proposed in this article by integrating traditional quantitative genetic theory and the latest discoveries of epigenetic effects will allow geneticists to chart a more comprehensive picture of the genetic landscape for complex phenotypes underlying agricultural production, physiology and human diseases.

## Competing interests

The authors declare that there are no competing interests.

## Authors’ contributions

ZW designed the algorithm and conducted the simulation experiments. ZHW derived the statistical model for hypothesis tests. JW participated in computer simulation. YHS JW participated in computer simulation. JZ provided biological insights for the statistical model. DL supervised the project. RW conceived of the model, designed the computer simulation and wrote the manuscript. All authors read and approved the final manuscript.
